# Assessment of Family Planning Counseling Provided for Postpartum Women and Associated Factors

**DOI:** 10.1155/2020/2649340

**Published:** 2020-01-25

**Authors:** Munit Abdulreshid, Hailemichael B. Dadi

**Affiliations:** ^1^Department of Medicine, Saint Paul's Hospital Millennium Medical College, Addis Ababa, Ethiopia; ^2^Epidemiology Unit, Department of Public Health, Saint Paul's Hospital Millennium Medical College, Addis Ababa, Ethiopia

## Abstract

**Background:**

Good quality family planning counseling particularly in the postpartum period is paramount to contraception adoption and continuation; it is also paramount in the reduction of maternal as well as infant morbidity and mortality.

**Objective:**

Assess the level of family planning counseling provided for women in their immediate postpartum period in the labor ward at Saint Paul's Hospital in 2019.

**Method:**

An institution-based cross-sectional study was conducted from February to March of 2019 among women in the labor ward. A face-to-face exit interview was conducted with 209 randomly selected women. A structured pretested questionnaire was used to assess the level of family planning counseling provided. Bivariate logistic regression was used to test for statistical association.

**Results:**

Only 28.2% of the counseling sessions were adequate. Most (58.9%) of the counseling sessions did not maintain the privacy of the client. In 67.9% of the sessions, the counselor did not ask the concern of the client regarding the use of modern family planning methods and 74.2% of the clients were not told about the possible side effects of a method. Clients with no formal education (OR = 2.52, 95%CI = 1.1‐3.3) and those with only primary level education (OR = 1.22, 95%CI = 1.04‐3.02) were more likely to have had inadequate counseling.

**Conclusion:**

The level of family planning counseling was inadequate. The study indicated the need to provide training for service providers on family planning counseling with the existing standard frameworks but also in consideration of the sociodemographic background of the client, particularly their educational status.

## 1. Background

Family planning has a positive influence on women's health by reducing pregnancy-related health issues; this is achieved by reducing the rates of unintended pregnancies and the need for unsafe abortion [1, 2]. Postpartum family planning specifically focuses on the prevention of unintended and closely spaced pregnancies through the first year following childbirth [2–4]. Closely spaced pregnancies within the first year postpartum increase risks for adverse outcomes, such as preterm and low birth weight [1].

The estimated unmet need for contraception for married women in sub-Saharan Africa is 27.7% [5–8]. In Ethiopia, according to the 2016 Ethiopian Demographic and Health Survey (EDHS), the use of modern contraceptives has increased from 6% in 2000 to 35% in 2016 [9]. Despite this progress, there are still multitudes of factors that influence informed choice by clients as well as satisfying clients' need for family planning.

Studies show that the quality of family planning counseling service influences contraception adoption, prevalence, and continuation [10–14]. And yet, the level of quality of family planning counseling is not well studied in the present study area. A few previous studies conducted in other study areas have identified a multitude of factors associated with the quality of family planning counseling [15–22]. However, most of these previous studies are aimed at assessing the quality of family planning counseling provided for women during prenatal care. However, studies show that the use of more effective methods of contraception is the highest when counseling is provided during both prenatal and postpartum time periods [1]. Therefore, the present study focused on assessing the level of family planning counseling provided for postpartum women and associated factors.

## 2. Methods

### 2.1. Study Design and Setting

This was an institution-based cross-sectional study conducted at Saint Paul's Hospital Millennium Medical College (SPHMMC) in Addis Ababa, the capital city of Ethiopia, conducted from February to March 2019. SPHMMC is the second biggest hospital in the country and provides medical specialty services to patients who are referred from all over the country. The hospital's labor ward has a patient flow of an average of 840 patients per month. In the ward, a number of services are provided, one of which is postpartum family planning counseling. The service providers include OBGYN residents, medical interns, and midwives. The source population for this study was women in their immediate postpartum period at SPHMMC's labor ward. The study population was randomly selected from among women in their immediate postpartum period at SPHMMC's labor ward. Women who had received postpartum family planning counseling were included in the study.

### 2.2. Sample Size and Sampling Technique

The sample size was calculated by the formula for a single population proportion for a cross-sectional survey taking a proportion of 42.9% from a similar study [18]. A confidence interval of 95% and a degree of precision of 5% were used. A contingency of 10% was added to the sample size for nonresponders. The patient flow over a month's period (i.e., *N* = population size) in the labor ward on average is 840 patients. The calculated sample size is 259, and by adding 10% for nonresponders, the total sample size is 285. After correcting for a finite population, the final sample size was 213.

Considering the patient flow over a month's period in the labor ward, a systematic random sampling technique with a sampling interval of three (*K* = *N*/*n* = 840/285 = 2.9 ≈ 3) was used to select study participants. The dependent variable in this study was the level of family planning counseling, and the independent variables were client characteristics: client's age, educational level, religion, and parity.

### 2.3. Operational Definitions

The level of family planning counseling was assessed based on adherence to a standard framework which includes the variables Greet, Ask, Tell, Help, Explain, and Return (GATHER).

#### 2.3.1. GATHER

*Greet* included 4 variables: provider giving greeting, welcoming gesture, addressing respectfully, and arranging for privacy. *Ask* included 6 variables: asking about medical history, desired fertility, previous use of family planning, client's interest in family planning, concerns about family planning methods, and the time that the client and her partner has spent living together. *Tell* included 5 variables: mentioning all family planning methods, asking about what is already known about the method, describing the effectiveness of the method, asking which method interests the client, and correcting of incorrect information. *Help* included 5 variables: involving the husband/partner in the consultation, inquiring about concerns on a method, responding to concerns, recommending a method, and discussing reasons why a method is not appropriate for the client. *Explain* included 5 variables: telling how to use the method, use of visual aids, mentioning side effects, teaching how to manage side effects, and asking the client to recite the information provided. *Return* included 2 variables: scheduling the next visit and informing when to come before the scheduled visit.

#### 2.3.2. Level of Family Planning Counseling

Family planning counseling is classified as “adequate” or “inadequate” counseling by adding up the scores (0s and 1s) from the GATHER items and adding up the total score ranging from 0 to 25. The total score is then categorized as “adequate” if ≥16 and “inadequate” if ≤15. In addition, scores of ≥24 are considered “excellent,” 19 to 23 “good,” 16 to 18 “average,” 11 to 15 “poor,” and ≤10 “very poor” [18].

### 2.4. Data Collection Tool and Procedure

Data was collected via a face-to-face exit interview of mothers in their immediate postpartum period in the labor ward. The questionnaire was a slightly modified adaptation of Young Mi Kim and Cheryl Lettenmaier's “Tools to Assess Family Planning Counseling: Observation and Interview” from the John Hopkins School of Public Health Center for Communication Programs [23]. The data collection was supervised by the principal investigator while data was collected by trained health professionals. The questionnaire was pretested on 5% of the sample size to test the clarity of questions.

### 2.5. Data Processing and Analysis

Data were checked and cleaned for completeness and errors in data entry before analysis. In order to identify factors associated with the level of family planning counseling, bivariable regression was implemented. A *p* value of 0.05 was used as a cutoff point to establish association.

### 2.6. Ethical Consideration

Ethical clearance was obtained from SPHMMC's Institutional Review Board. Informed verbal consent was received from all participants of the study after being informed regarding the purpose of study.

## 3. Results

### 3.1. Sociodemographic Characteristics

Of 213 women approached, 209 (98.1%) were willing to be interviewed. Of the 209 respondents, majority (84.3%) of the respondents were aged 19 to 35 years. Most (41.6%) of the respondents had no formal education and 90.4% were married (Table 1).

### 3.2. Level of Family Planning Counseling

Most (58.9%) of the counseling sessions did not maintain the privacy of the client during the consultation. Most (74.2%) of the clients were not told about the possible side effects related to the use of a method (Table 2).

### 3.3. Factors Associated with Level of Counseling

Only 59 (28.2%) of the counseling sessions were adequate, while 59 (28.2%) were inadequate, 131 (62.8%) were poor, 19 (9.1%) were very poor, 35 (16.7%) were average, and only 24 (11.5%) were “good”; one of the sessions was regarded as “excellent.” Variables such as the sociodemographic characteristics of the clients (such as age, parity, educational status, religion, and marital status) and time of counseling were entered into bivariate analysis. The educational status of the clients was statistically associated with the level of family planning counseling. Clients having no formal education (COR = 2.52, 95%CI = 1.1‐3.3) and those having only primary level education (COR = 1.22, 95%CI = 1.04‐3.02) were more likely to report inadequate counseling sessions (Table 3).

## 4. Discussion

This study assessed the level of family planning counseling services and factors associated with the clients' perspective. Only 28.2% of the family planning counseling sessions in the labor ward were adequate. This is significantly lower than a similar study done in clinics in Jordan (42.9%) [18] as well as in a study done in urban primary health care facilities in Iran (38.9%) [19]. But it was higher than a study conducted in health facilities of Senegal (18%) [20]. The difference observed might be explained by the disparity in facility infrastructure, provider training and experience, and the overall health service delivery system. This study was done in the labor ward where family counseling is only part of other major services given; however, in other studies [18, 19], the setting was in clinics where the primary focus of providers is in delivering family counseling services.

Regarding the specific component of the standard framework of family planning counseling, significant disparities were observed between this and other similar studies. Despite the paramount importance of male involvement in family planning uptake, the assessment of the level of involvement of the client's partner during consultation was lower than that of a study conducted in Iran (39.7% vs. 50.5%) [19]. Furthermore, the counseling sessions were not arranged in a way that maintains the privacy of the client mainly due to a lack of adequate counseling room setup.

The level of proactively exploring the concerns of clients regarding the use of modern methods was also lower (32.5%) than that of a similar study (100%) [18]. Studies show that clients' lack of opportunity to ask questions and clarify doubts during counseling were a major source of client dissatisfaction [24]. The reason for this poor provider-client communication could be rooted in a multitude of factors including mainly inadequate provider knowledge and/or a lack of training in communication and counseling skills, poor job satisfaction, poor working conditions, and workload [19].

In this study, only 25.8% and 14.8% of service providers informed their clients about the side effects related to the use of a family planning method and how to deal with these side effects, respectively. This was significantly lower than the level reported in a similar study—67.2% and 56.3%, respectively [19]. A possible explanation for this might be that service providers in this study purposely withheld information to reduce the refusal rate for use of modern methods by not emphasizing on the side effects. Or it could have been due to poor provider knowledge concerning contraceptive side effects, which is a common problem in developing countries [19].

Women with no formal education or with only primary education were more likely to have received inadequate counseling service. This finding highlights the gap in the provision of counseling that is suitable for clients with lower educational levels. Alternatively, a possible reason for this finding could be that this study utilized only a client's interview to assess the level of counseling which is limited to the perception of the client.

One of the possible limitations of this study is that it attempted to assess the level of counseling from the clients' perspective while a relatively objective approach would have been a direct observation of the counseling sessions. Moreover, the crude analysis method implemented is not suited to rule out possible confounding factors.

## 5. Conclusion

The overall level family planning counseling provided for postpartum women was inadequate. The findings of this study suggest that the counseling sessions need improvement in terms of provider-client communication. Moreover, the counseling sessions should be arranged to maintain the privacy of the clients and ensure the involvement of the client's partner. Formal training should be provided for service providers on family planning counseling designed based on existing standard frameworks but also considering the sociodemographic background of the clients particularly their educational status.

## Figures and Tables

**Table 1:** Characteristics of women in SPHMMC's labor ward, Addis Ababa, Ethiopia, 2019.

Variables	Frequency (%)
Age	
<19	7 (3.3)
19-35	176 (84.3)
>35	26 (12.4)
Religion	
Orthodox	130 (62.2)
Muslim	45 (21.5)
Protestant	34 (16.3)
Educational level	
No formal education	87 (41.6)
Primary	55 (26.3)
Secondary	50 (23.9)
Higher education	17 (8.1)
Marital status	
Single	13 (6.2)
Married	189 (90.4)
Divorced or widowed	7 (3.4)

**Table 2:** Level of family planning counseling utilizing the GATHER framework, Addis Ababa, Ethiopia 2019.

Variables	Response	
	Yes, *N* (%)	No, *N* (%)
Greet		
Greet the client?	206 (98.6)	3 (1.4)
Make welcoming gestures?	144 (68.9)	65 (31.1)
Address respectfully?	206 (98.6)	3 (1.4)
Arrange for privacy	86 (41.1)	123 (58.9)
Ask		
Ask client's medical history	208 (99.5)	1 (0.5)
Ask how much time the client and her partner lived together	40 (19.1)	169 (80.9)
Ask client's plan to have more children	144 (54.5)	95 (45.5)
Ask if client has used family planning methods before	149 (71.3)	60 (28.7)
Ask if client is interested in using modern methods	153 (73.2)	56 (26.8)
Ask if client was concerned about using modern methods	67 (32.5)	142 (67.9)
Tell		
Mention all methods of family planning available	0	209 (100)
Ask what is already known about the method	64 (30.6)	145 (69.4)
Describe the effectiveness of methods	88 (42.1)	121 (57.9)
Ask the preferred method	74 (35.4)	135 (64.4)
Clear up myths/rumors/incorrect information	140 (67)	69 (33)
Help		
Ask about partner's attitude towards using a method	83 (39.7)	126 (60.3)
Ask client's worries about using modern methods	68 (32.5)	141 (67.5)
Acknowledge and respond to client's concerns about using a method	50 (23.9)	159 (76.1)
Discuss the reason why some methods might not be appropriate for the client	169 (80.9)	40 (19.1)
Recommend a specific method	206 (98.6)	3 (1.4)
Explain		
Explain how to use a method	126 (60.3)	68 (939.7)
Use visual aids to help explain the method	2 (1)	207 (99)
Explain possible side effects related to use	54 (25.8)	155 (74.2)
Explain how to deal with possible side effects	31 (14.8)	178 (85.2)
Ask to repeat important instructions	19 (9.1)	190 (90.9)
Return		
Schedule a follow-up appointment	206 (98.6)	3 (1.4)
Tell client to come back before appointment if there are any problems	197 (94.3)	12 (15.7)

**Table 3:** Level of family planning counseling, Addis Ababa, Ethiopia 2019.

	Level of counseling			
	Inadequate (%)	Adequate (%)	COR, 95% CI	*p* value
Age				
Less than 19 years	6 (85.2%)	1 9 (14.8%)	1.8 (0.43-2.67)	0.105
19 to 35 years	134 (76.1%)	42 (23.9%)	2.83 (0.74-3.23)	
More than 35 years	9 (34.6%)	17 (65.4%)	1	
Religion				
Protestant	25 (73.5%)	9 (26.5%)	0.91 (0.38-2.1)	0.824
Muslim	27 (60%)	18 (40%)	0.49 (0.24-1.04)	
Orthodox	98 (75.4%)	32 (24.6%)	1	
Educational level				
No formal education	77 (88.5%)	10 (11.5%)	2.52 (1.1-3.4)	0.001^∗^
Primary education	44 (80%)	11 (20%)	1.22 (1.04-3.02)	
Secondary education	24 (48%)	26 (52%)	3.25 (0.93-5.03)	
Higher education	4 (23.5%)	13 (76.5%)	1	

^∗^ *p* value < 0.05; COR: crude odds ratio.

## Data Availability

The data used to support the findings of this study are available from the corresponding authors upon request.
